# Associations of parathyroid hormone and atrial fibrillation: epidemiological insights from the LURIC study

**DOI:** 10.3389/fcvm.2026.1885117

**Published:** 2026-07-08

**Authors:** L. König, M. R. Grübler, N. Verheyen, E. Kolesnik, M. Wallner, A. J. van Ballegooijen, D. von Lewinski, S. Pilz, A. Tomaschitz, W. März

**Affiliations:** 1 Medical University of Vienna, Vienna, Austria; 2 Department of Internal Medicine with Cardiology, Nephrology and Intensive Care Medicine, Universitaetsklinikum Wiener Neustadt, Wiener Neustadt, Austria; 3 Medical Faculty, Sigmund Freud University, Vienna, Austria; 4 Department of Medicine, Faculty of Medicine and Dentistry, Danube Private University, Krems, Austria; 5 Department of Cardiology, Medical University Graz, Graz, Austria; 6 Department of Epidemiology and Biostatistics, EMGO Institute for Health and Care Research, Vanderbilt University Medical Centre, Amsterdam, Netherlands; 7 Department of Health Sciences and the EMGO Institute, Vanderbilt University Amsterdam, Amsterdam, Netherlands; 8 Department of Internal Medicine, Division of Endocrinology and Metabolism, Medical University of Graz, Graz, Austria; 9 Clinical Institute of Medical and Chemical Laboratory Diagnostics, Medical University of Graz, Graz, Austria; 10 Synlab Academy, Synlab Services LLC, Mannheim, Germany; 11 Vth Department of Medicine (Nephrology, Hypertensiology, Rheumatology, Endocrinology, Diabetology, Lipidology), Medical Faculty Mannheim, University of Heidelberg, Mannheim, Germany

**Keywords:** atrial fibrilation, parathyroid hormone, stroke LURIC, epidemiology

## Abstract

**Introduction:**

Parathyroid hormone (PTH) influences calcium handling in cardiomyocytes and sympathetic cardiac signaling. It has been linked to arterial hypertension and cardiovascular mortality. We hypothesized that PTH is independently associated with atrial fibrillation (AF).

**Methods:**

We analyzed data from 3,201 patients (mean age 63 ± 13 years, 25.8% female) enrolled in the LUdwigshafen RIsk and Cardiovascular health (LURIC) study. We defined AF based on medical history or current ECG. Multivariable binary logistic regression was used to assess the association between PTH and AF, adjusting for established AF risk factors, parameters of mineral metabolism, kidney function, and concomitant medication.

**Results:**

AF was present in 389 patients (12.2%). PTH was significantly associated with AF in the primary multivariable model [OR 3.96 (2.32–6.76), *p* < 0.001]. In the fully adjusted model including mineral metabolism and medication variables, the association remained significant [OR 2.32 (1.24–4.35), *p* = 0.009]. The association remained significant after additional adjustment for markers of cardiac dysfunction, including invasive hemodynamic parameters.

**Conclusion:**

Elevated PTH was associated with prevalent AF in patients referred for coronary angiography after adjustment for common confounders. The association persisted after adjustment for structural, neurohumoral and invasive hemodynamic markers, suggesting that elevated PTH is not merely a surrogate of cardiac dysfunction but may represent an independent correlate of AF. Prospective studies are needed to clarify the temporal and causal relationship between PTH and AF.

## 1 Introduction

High serum levels of parathyroid hormone (PTH), even within the normal range, are independently associated with increased cardiovascular and all-cause mortality (1, 2). This might be partially explained by pro-arrhythmogenic effects of PTH (3). Experimental and translational data suggest that PTH exerts direct effects on cardiomyocytes via PTH1 receptor signaling, including activation of intracellular calcium pathways (4, 5) and interaction with the renin–angiotensin–aldosterone system (6). These mechanisms have been summarized in recent reviews, which also highlight potential electrophysiological effects of PTH including alterations in repolarization and potential pro-arrhythmogenic effects (3, 7). Concordantly, in clinical studies elevated PTH within the normal range predicted sudden cardiac death (8). Patients with primary hyperparathyroidism (pHPT) exhibit an increased electrical vulnerability which is reversible by parathyroidectomy (9).

In patients with atrial fibrillation, PTH was related to left atrial size (10). Nevertheless, it should be taken into consideration that, particularly in AF, maintenance of serum PTH is due to complex mechanisms involving parameters of calcium metabolism, drugs and co-morbidities. Besides the interference with serum calcium and 25-hydroyvitamin D_3_ (VitD3), serum PTH is affected by digitalis, calcium antagonists and diuretics which are commonly used drugs in patients with AF or in concomitant co-morbidities (11–13).

Although elevated PTH has previously been associated with AF, prior reports did not account for the complex interplay of mineral metabolism, renal function and concomitant medication that jointly determine circulating PTH; whether the association ins independent of these factors therefore remains unsolved. Hence, we aimed to test the hypothesis that PTH is associated with AF even after consideration of comorbidities, parameters of calcium metabolism and drug intake as potential confounders in a large cohort of patients referred to coronary angiography.

## 2 Methods

### 2.1 Participants and study design

The design of the LUdwigshafen RIsk and Cardiovascular Health (LURIC) study has been reported in detail elsewhere (14). Briefly, LURIC is a prospective study on patients (*n* = 3,316) referred to coronary angiography in a tertiary care center in Germany between July 1997 to January 2000. Baseline examinations were performed on the day of admission to coronary angiography. Inclusion criteria were Caucasian ancestry and clinical stability except for acute coronary syndrome (ACS). For the present analysis we excluded patients with no serum PTH measurement (*n* = 84) or no data on the history of AF if the current ECG showed no AF (*n* = 32). ECG data were available in all patients. The final sample consisted of 3,201 patients. Stroke outcomes were systematically assessed during scheduled follow-up visits, analysis was restricted to the first 7.5 years, the period for which structured outcome data were available.

The study was approved by the ethics committee of the Ärztekammer Rheinland-Pfalz, Germany and all study participants gave their written informed consent. The LURIC study is in concordance with the Declaration of Helsinki.

### 2.2 Laboratory measurements, ECG and echocardiography

All laboratory measurements, 12-lead ECG and echocardiography were performed in the morning before coronary angiography. All patients with AF in the present ECG or a history of AF were classified as AF patients. A detailed description of baseline examinations has already been published (14). Routine parameters used in our analysis were determined in serum after blood sampling and all other parameters, including PTH, 25-hydroxy vitamin D_3_and pro-brain natriuretic peptide (NT-proBNP) were measured from blood specimen which had been frozen immediately after blood sampling and stored at −80 °C until use for laboratory measurements.

The serum concentrations of intact PTH were determined by ElectroChemiLuminescence Immunoassay (ECLIA) on an Elecsys 2010 (Roche Diagnostics, Mannheim, Germany). The reference ranges for PTH were 15–65 pg/mL, with an inter-assay variation of 5.7%–6.3%.

Left ventricular ejection fraction was determined during coronary angiography and was qualitatively assessed into normal, slightly impaired, moderately impaired or severely impaired.

### 2.3 Statistical methods

Continuous variables are expressed either as mean +/- standard deviation (SD) for parametrically distributed or median +/- interquartile range (IR) for not normal distributed parameters and categorical variables as number and percentages. All variables without normal distribution were transformed logarithmically (10log) before use in parametric calculations except for age where we calculated with age squared.

We calculated Pearson or Spearman correlation of PTH with AF risk factors including mean systolic blood pressure left atrial diameter, left ventricular (LV) function and CAD. We used one-way ANOVA to assess the correlation of PTH with the number of prescribed antihypertensive drugs and extent of coronary artery disease. Baseline differences between groups (AF vs. no AF) were assessed by one-way ANOVA for continuous parameters and either Chi-squared test or Fisher's exact test for categorical parameters with *p* for 2-sided tests or for linear-by-linear association. For comparison between these groups with continuous covariates not distributed parametrically we applied either Mann–Whitney-U Test or Kruskal–Wallis Test depending on the number of strata of the dependent variable.

Binary logistic regression analysis with AF as the dependent variable was performed using a hierarchical model. PTH was log10-transformed to account for skewed distribution prior to regression-analysis, odds ratios reflect the increase in odds of AF per 10-fold increase in serum PTH concentrations. For clinical interpretability, odds ratios per doubling of PTH are additionally reported for the primary and fully adjusted models (Table 3).

Model 1 included PTH, age and sex. Model 2 additionally included established risk factors for AF [low density lipoprotein cholesterol (LDL), thyroid stimulating hormone (TSH), type 2 diabetes, systolic office blood pressure, mean heart rate, history of myocardial infarction and coronary artery disease (CAD)]. In model 3a we added estimated glomerular filtration rate (eGFREPI), 25-hydroxy vitamin D_3_, serum calcium, serum phosphate and fibroblast growth factor 23 (FGF23) to model 2. Model 3b included calcium antagonists and digitalis 2; Model 3c added diuretics, ACE inhibitors, ARBs, beta blockers, statins, aspirin, heparin and vitamin K antagonist. In model 4 we included all parameters of model 3a, 3b and 3c. Medication variables were included in secondary models; however, as some treatments (e.g., digitalis, anticoagulation) are frequently initiated as a consequence of AF, adjustment for these variables may introduce collider bias. Therefore, medication-adjusted analyses were considered exploratory.

To explore whether the association between PTH and AF was independent of cardiac dysfunction and hemodynamic burden, two pre-specified sensitivity analyses were performed. First, in a subset of patients with available invasive hemodynamic data, additional adjustment for catheter derived LVEF, cardiac index, left ventricular end-diastolic pressure, and mean pulmonary artery pressure was performed. Second, in the full cohort, additional adjustment for NYHA functional class, NT-proBNP and left atrial diameter was applied. These analyses were considered exploratory given their sensitivity analysis character and the risk of overadjustment if these variables act as mediators rather than confounders. Multicollinearity was assessed using variance inflation factors (VIF), with values >5 considered indicative of relevant collinearity. Due to collinearity between wedge pressure and mPAP, wedge pressure was excluded from the final invasive model. Effect estimates are reported as odds ratios (OR) with 95% confidence intervals (CI). A two-sided *p*-value <0.05 was considered statistically significant.

To understand the relevance of potential confounders, we repeated the regression analysis using model 2 in subgroups of patients with or without arterial hypertension (yes/no), and in further analysis, we split the cohort according to the serum levels of FGF23 and aldosterone in two groups according to the median.

Given that PTH has been associated with endothelial dysfunction and subclinical atherosclerosis, and that AF itself predisposes the thromboembolism, we additionally explored whether elevated PTH translates into an increased downstream risk of stroke (15, 16). The association between PTH and time to stroke was examined using Cox proportional hazards regression models. Results are presented as hazard ratios (HR) with corresponding 95% confidence intervals (CI). Multivariable models were adjusted for age, sex and AF status. Stroke events were systematically assessed during follow-up visits up to 7.5 years after baseline. As no structured data on stroke occurrence were available beyond this time point, analysis of stroke outcomes was restricted to the first 7.5 years of follow-up. PTH was log10-transformed prior to analysis; hazard ratios represent the increase in hazard of stroke per 1 log10-unit increase in PTH.

The SPSS version 27.0 was used to perform statistical analyses (SPSS Inc., Chicago). A *P*-value < 0.05 was considered statistically significant. Differences in sample size across models reflect missing data in mineral metabolism and medication variables; complete case analysis was applied.

### 2.4 Definitions

We applied the following definitions for comorbidities. Type 2 diabetes mellitus was diagnosed if patients had a history of type 2 diabetes or if the diagnostic criteria published by the ADA 2025 were met (17). Active and ex-smokers were classified as smokers. Coronary artery disease was diagnosed if patients had at least one visual stenosis of > 50% or a significant stenosis of at least one major vessel as assessed by coronary angiography. Extent of CAD was defined as 0 for no stenosis > 49% in any major vessels and 1–3 for the number of major vessels with a stenosis of > 49%. Systolic heart failure was defined in case of an impaired left ventricular function based on visual assessment during coronary angiography.

Time-to-event was defined as the interval between baseline and the first occurrence of stroke (whether ischemic or haemorrhagic) or censoring. Patients who did not experience a stroke within 7,5-year follow-up period were administratively censored at 7,5 years. Patients who died without prior stroke were censored at the time of death.

## 3 Results

### 3.1 Baseline characteristics

Both ECG and serum concentrations of PTH were available in 3,201 patients. The mean age was 63 years ± 13 years and 827 were females (25.8%). We defined 389 (12.2%) patients with AF (110 females, 28.3%) with a mean age of 66 ± 9 years. Of those 370 (95%) had a positive history of AF and 155 (40%) had AF in the present ECG, and 135 (35%) had AF in both their medical history and the current ECG.

**Figure 1:**
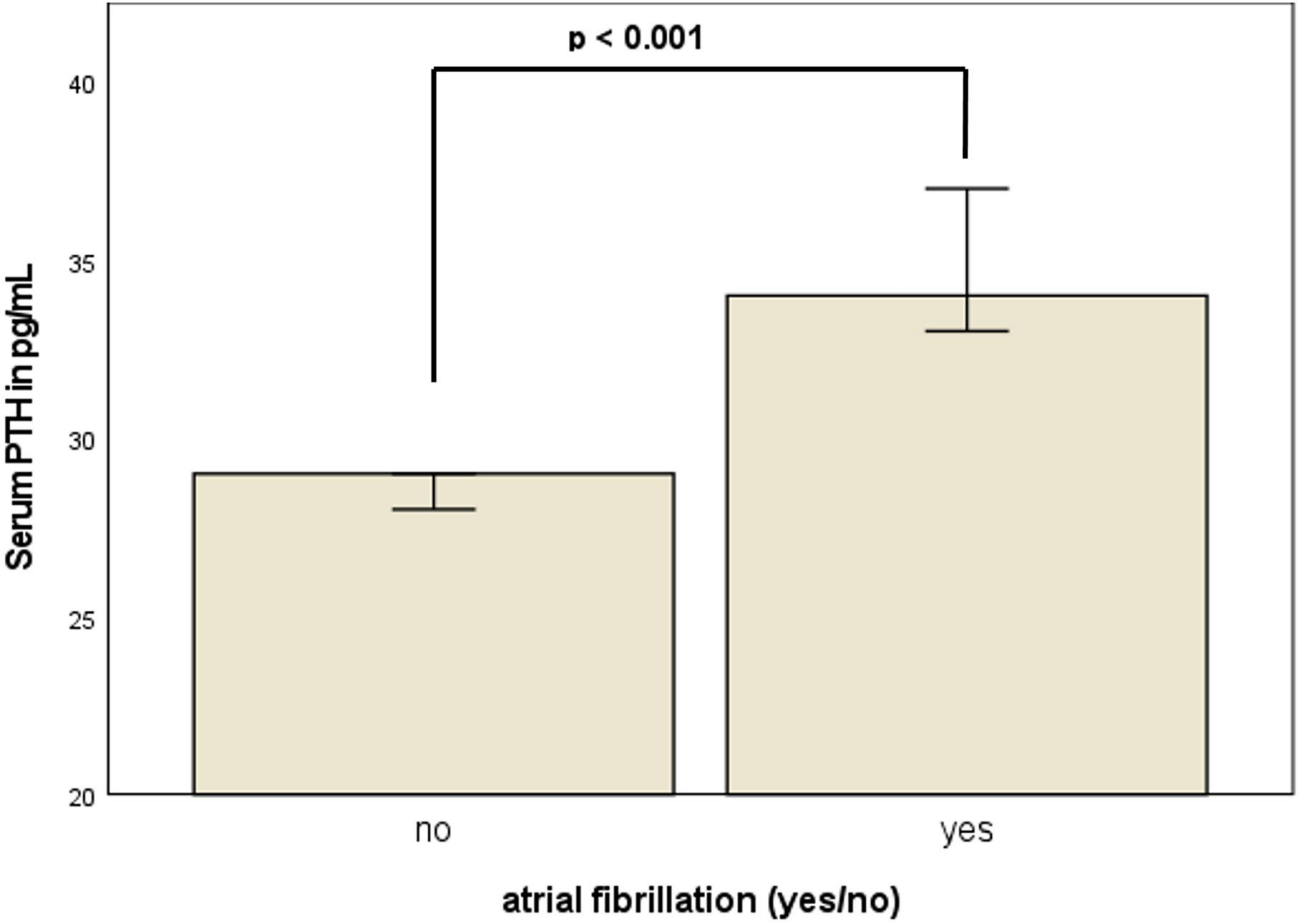
Serum levels of parathyroid hormone (PTH) in patients without AF vs. patients with AF. The difference between the groups was assessed with Mann–Whitney-U test.

Serum PTH was significantly higher in patients with AF compared to those without AF [median (IR) 34 (25–46) vs. 29 (21–38), *p* < 0.001, Figure 1] and did not differ between patients with and without AF in the current ECG (*p* = 0.396). Patients with AF were older, had a lower prevalence of CAD and history of MI (*p* < 0.001, respectively) and were more often prescribed ACE-inhibitors, antiplatelet agents, calcium antagonists, digitalis, diuretics, vitamin K antagonist and heparin (*p* < 0.01, respectively) and less often beta-blockers and statins (*p* < 0.01, respectively). They had lower serum 25-hydroxy vitamin D_3_, GFREPI and were taking more antihypertensive drugs (*p* < 0.01, respectively). No differences were observed in systolic or diastolic blood pressure (*p* = 0.66 and *p* = 0.69, respectively). Baseline characteristics of all groups are illustrated in Table 1.

**Table 1:** Baseline characteristics.

*n* = 3,201	No AF (*n* = 2,812, 88%)	AF (*n* = 389, 12%)	*p*-value
Basic demographics			
Age, years (IQR)	62 (51–73)	66 (57–75)	<0.001
Female Sex, n%	827 (25%)	110 (28%)	0.378
Systolic blood pressure, mmHg (IQR)	141 (124–156)	140 (123–160)	0.810
Mean heart rate, bpm (IQR)	67 (60–75)	67 (59–76)	0.488
BMI, kg/m^2^ (IQR)	27.5 (23.2–31.6)	27.6 (23.3–31.5)	0.557
Comorbidities			
Coronary artery disease, n%	1,911 (70%)	219 (56%)	<0.001
History of myocardial infarction, n%	1,165 (43%)	119 (30%)	<0.001
Type 2 Diabetes mellitus, n%	1,071 (39%)	174 (44%)	0.610
History of arterial hypertension, n%	1,594 (58%)	238 (60%)	0.444
Smoking status, n%	1,766 (65%)	240 (61%)	0.160
NYHA class, n%			
I	1,452 (53%)	170 (43%)	<0.001
II	800 (29%)	111 (28%)	
III	402 (15%)	96 (24%)	
IV	78 (3%)	17 (4%)	
Medication			
Intake of ACE-inhibitors, n%	1,433 (52.5%)	233 (59%)	0.13
Intake of ARBs, n%	114 (4%)	27 (7%)	0.26
Intake of antiplatelet agents, n%	2,070 (76%)	169 (43%)	<0.001
Intake of beta blockers, n%	1,798 (66%)	193 (49%)	<0.001
Intake of calcium antagonists, n%	377 (14%)	104 (26%)	<0.001
Intake of statins, n%	1,339 (49%)	147 (37%)	<0.001
Intake of digitalis, n%	280 (10%)	200 (51%)	<0.001
Intake of diuretic, n%	697 (25.5%)	191 (48.5%)	<0.001
Intake of vitamin K- antagonists, n%	94 (3%)	114 (29%)	<0.001
Intake of heparin, n%	665 (24%)	158 (40%)	<0.001
Laboratory parameters			
Parathyroid hormone, pg/mL	29 (21–38)	34 (25–46)	<0.001
25-hydroxy vitamin D_3_, µg/L	16 (10–23)	14.5 (9–21)	0.012
Serum calcium, mmol/L (IQR)	2.33 (2.20–2.36)	2.33 (2.18–2.48)	0.554
Serum phosphate, mg/dL (IQR)	3.5 (3.1–3.9)	3.5 (3.1–3.9)	0.491
eGFR_EPI_, mL/min/1.73 m² (IQR)	80 (62.5–97.5)	74.5 (55.5–93.5)	<0.001
LDL cholesterol, mg/dL (IQR)	115 (95–138)	109.5 (87–137)	0.003
TSH, mU/L (IQR)	1.24 (0.77–1.91)	1.18 (0.64–1.81)	0.019
NT-proBNP pg/mL (IQR)	254 (98–708)	858.5 (285–2,075)	<0.001
FGF23 RU/mL (IQR)	103 (68–138)	301 (232–370)	<0.001

Baseline differences between patients with no AF and patients with AF. Continuous variables are either expressed as mean ± standard deviation or median (interquartile ranges) and categorical variables as number and percentages. For continuous variables, the difference between the groups was assessed with either Mann–Whitney-U test or student's t-test. For categorical variables Chi-squared-test was applied.

PTH significantly correlated with age (Pearson r = 0.217, *p* < 0.001), systolic blood pressure (r = 0.091, *p* < 0.001), left atrial diameter (r = 0.150, *p* < 0.001), LV function (r = −0.134, *p* < 0.001) and increased with the number of prescribed antihypertensive drugs (*p* between groups=0.013), but not with the extent of CAD (*p* = 0.707). PTH was significantly higher in patients taking calcium antagonists (36.8 pg/mL ± 23 vs. 33.6 pg/mL ± 29.7, *p* < 0.001) or digitalis (42 pg/mL ± 31, 32.7 pg/mL ± 27.9, *p* < 0.001), respectively. Results are presented in Table 2.

**Table 2:** Correlations of serum PTH with baseline parameters.

Parameter	Method	Result	*p*-value
Age	Pearson r	0.217	<0.001
Systolic blood pressure	Pearson r	0.091	<0.001
Left atrial diameter	Pearson r	0.150	<0.001
LV function	Pearson r	−0.134	<0.001
No. of antihypertensive drugs	One-way ANOVA	increasing PTH across groups	0.013
Extent of coronary artery disease	One-way ANOVA	no association	0.707
Calcium antagonists (yes vs. no)	Group comparison	36.8 ± 23 vs. 33.6 ± 29.7	<0.001
Digitalis (yes vs. no)	Group comparison	42 ± 31 vs. 32.7 ± 27.9	<0.001

Associations of serum parathyroid hormone (PTH) with baseline parameters. PTH was log10-transformed for Pearson correlations (r). Associations with the number of prescribed antihypertensive drugs and the extent of coronary artery disease were assessed by one-way ANOVA. PTH levels by medication use are given as mean ± SD (pg/mL).

### 3.2 Regression analysis

Variables for the fully adjusted model were available in 2,613 cases of whom 389 had AF (12.2%). In model 1 of the binary logistic regression analysis PTH was significantly associated with AF [OR (95% CI) 4.67 (2.79–7.82), *p* < 0.001]. This correlation remained similar after adjustment for risk factors for AF [model 2; OR 3.96 (2.32–6.76), *p* < 0.001] and parameters of calcium metabolism [model 3a: OR 2.42 (1.30–4.49), *p* = 0.005]. After adjustment for prescription of calcium antagonists and for digitalis the results remained stable [model 3b: OR 2.66 (1.30–4.49), *p* = 0.001]. Further adjustment for prescribed drugs did not materially alter these results [model 3c: OR 2.71 (1.51–4.89), *p* = 0.001]. In the fully adjusted model parameters significantly associated with AF were age, male sex, TSH, LV function, extent of CAD and FGF23, as well as prescription of digitalis, calcium antagonists, aspirin, vitamin K antagonists and heparin. The association of PTH and atrial fibrillation remained significant after full adjustment in the final model [model 4: OR 2.32 (1.24–4.35), *p* = 0.009]. Expressed per doubling of serum PTH, the corresponding odds ratios were 1.51 (1.29–1.78), *p* < 0.001 in Model 2 and 1.29 (1.07–1.57), *p* = 0.009 in the fully adjusted model (Model 4). Detailed results are given in Table 3. Results remained similar when excluding patients with pHPT from the analyses (*n* = 3, results not shown).

**Table 3:** Regression models.

Model	n AF (% of all cases)	Adjustments	SE	OR (95% CI)	*p*-value
Model 1	389 (12.2)	sex, age	0.263	4.67 (2.79–7.82)	<0.001
Model 2	389 (12.2)	+ AF risk factors	0.273	3.96 (2.32–6.76)	<0.001
Model 3a	330 (10.3)	+ mineral metabolism	0.316	2.42 (1.30–4.49)	0.005
Model 3b	361 (11.2)	+ AADs	0.295	2.66 (1.49–4.74)	0.001
Model 3c	361 (11.2)	+ other medication	0.300	2.71 (1.51–4.89)	0.001
Model 4	330 (10.3)	all parameters included	0.320	2.32 (1.24–4.35)	0.009

Results of the multivariate binary logistic regression analysis with atrial fibrillation (AF) as the dependent variable and serum levels of parathyroid hormone (PTH) as the independent variable. PTH was log10-transformed prior to analysis, odds ratios for AF per log10-unit increase in PTH are presented. For clinical interpretability, odds ratios per doubling of serum PTH were 1.51 (1.29–1.78) for Model 2 and 1.29 (1.07–1.57) for Model 4.

Model 1: PTH and adjustment for age and sex.

Model 2: model 1 and additional adjustment for LDL cholesterol, thyroid stimulating hormone, diagnosis of type 2 diabetes, systolic office blood pressure, mean heart rate, history of myocardial infarction, and coronary artery disease.

Model 3a: model 2 and additional adjustment for estimated glomerular filtration rate according to the EPI formula and 25-hydroxy vitamin D_3_, serum calcium, serum phosphate and FGF23.

Model 3b: model 2 and additional adjustment for intake of calcium antagonists and digitalis.

Model 3c: model 2 and additional adjustment for diuretics, ACE inhibitors, ARBs, beta blockers, statins, aspirin, heparin and vitamin K antagonists.

Model 4: model 2 and all additional parameters from models 3a-3c.

In two pre-specified sensitivity analyses exploring the potential role of cardiac dysfunction as a confounder, the PTH–AF association remained largely unchanged, results are presented in Table 4. First, invasive hemodynamic measurements were available in 352 patients, of whom 129 (36.6%) had AF. After adjustment for catheter derived LVEF, cardiac index, left ventricular end-diastolic pressure, and mean pulmonary artery pressure, PTH remained significantly associated with AF [OR 3.09 (1.27–7.53), *p* = 0.013]. Second, in the full cohort, after additional adjustment for NYHA functional class, NT-proBNP and left atrial diameter the association remained significant [OR 2.14 (1.05–4.38), *p* = 0.037].

**Table 4:** Association between PTH and AF in sensitivity analyses additionally adjusting for markers of cardiac dysfunction and hemodynamics burden.

Adjustment	Cases of AF included (% of all cases)	SE	OR (95% CI)	*p*-value
Model 2	389 (12.2)	0.273	3.96 (2.32–6.76)	<0.001
Invasive	129 (4.2)	0.455	3.09 (1.27–7.53)	0.013
Structural/neurohumoral	243 (7.6)	0.365	2.14 (1.05–4.38)	0.037

Similar to models 3a-c, model 2 was used as baseline and adjusted for available invasive and noninvasive parameters.

Invasive hemodynamic included angiography-derived LVEF determined by angiography, Cardiac Index, mPAP, and LVEDP measured via intracardiac manometry. Parameters sourced from clinical assessment (NYHA) and sonographic evaluation (LA-Diameter, echocardiography-derived LVEF) and laboratory analysis (NT-proBNP) were used in a second, separate model.

PTH was log10-transformed prior to analysis, odds ratios for AF per log10-unit increase in PTH are presented.

### 3.3 Subgroup analyses

All subgroup analyses were performed using model 2 of the regression analysis. The association between PTH and AF remained significant when including only those without AF in the current ECG [*n* = 155; OR 2.33 (1.11–4.92), *p* = 0.026] and trended towards significance when including only those with AF in the current ECG [*n* = 234; OR 2.25 (0.93–5.42), *p* = 0.072]. When stratifying the cohort by median age, the association between PTH and AF remained significant in both strata [above vs. below median age: OR 6.79 (3.62–12.74), *p* < 0.001 vs. OR 3.36 (1.29–8.77), *p* = 0.013]. When splitting the whole cohort according to the history of hypertension the relation remained significant. [HT vs. no HT: OR 2.83 (1.31–6.11), *p* = 0.008 vs. 1.56 (0.57–4.31), *p* = 0.388]. Moreover, PTH was only associated with AF in those patients above the median serum level of FGF23 (54.15 RU/mL) while the correlation coefficient remained similar [below vs. above FGF23 median: OR 2.55 (0.81–8.09), *p* = 0.111 vs. OR 2.22 (1.07–4.62), *p* = 0.033]. There was no difference of the relation when splitting the cohort according to the median serum level of aldosterone [below vs. above aldosterone median: OR 2.13 (0.88–5.13), *p* = 0.093] vs. OR 2.32 (0.92–5.85), *p* = 0.075).

### 3.4 Time-to-event analysis

In this cohort, PTH was not associated with stroke risk in either univariable [HR 1.50 (0.42–5.39), *p* = 0.531] or multivariable Cox regression analysis [adjusted HR 1.53 (0.40–5.88), *p* = 0.536]. Similarly, categorization of PTH based on the median did not reveal a significant association with stroke risk [multivariable, HR 1.29 (0.77–2.18), *p* = 0.339]. The lack of association may reflect the relatively low number of stroke events (*n* = 60), or alternatively, a true absence of effect of PTH on stroke risk.

## 4 Discussion

In our analysis of 3,201 patients referred to coronary angiography we observed that higher serum concentrations of parathyroid hormone (PTH) were statistically associated with the presence of atrial fibrillation (AF). This association persisted after adjustment for established AF risk factors, markers of cardiac dysfunction, kidney function, parameters of mineral metabolism, and cardiovascular comorbidities. In contrast, PTH was not associated with incident stroke during follow-up.

Various prospective studies by our group and others and one recent meta-analysis consistently implicate that PTH is an independent predictor of cardiovascular and all-cause mortality (2, 8). Recent studies aimed to explain this increased PTH-related risk by its close interrelationship with congestive heart failure (HF) and arterial hypertension, respectively (18, 19). Although both of these cardiovascular morbidities represent major risk factors for the development of cardiac arrhythmias, few studies addressed the potential link between PTH and atrial fibrillation. Our findings are consistent with a previous report demonstrating higher PTH levels in patients with early-onset AF compared to controls (10). In that study, patients with persistent AF had higher PTH levels than those with paroxysmal AF. However, this retrospective, cross-sectional analysis was limited by the fact that neither drug intake, kidney function nor serum VitD levels as potential mediators of PTH excess were taken into account in the analysis. Furthermore, as PTH is a major regulator of bone mass density we consider it consistent with our findings that in patients with HF particularly those with AF are at increased risk of bone frailty and fractures (20–22).

Several mechanisms may account for the observed association between elevated PTH and AF. PTH receptors (PTH1R) are expressed in both ventricular and atrial cardiomyocytes, where PTH has been shown to augment norepinephrine release via cAMP-dependent signalling and increases intracellular calcium load through enhanced transmembrane influx and sarcoplasmic reticulum leak via ryanodine receptors (4, 5, 7, 23). As sympathetic overactivation and sarcoplasmic reticulum calcium mishandling are both established triggers of atrial arrhythmogenesis, these pathways represent plausible mechanistic candidates linking PTH to AF, though direct evidence from interventional studies is lacking (24, 25).

Second, the possibility of reverse causation should be considered. Given that AF is associated with upregulation of the renin–angiotensin–aldosterone system (RAAS), secondary hyperparathyroidism as a consequence of aldosteronism might be proposed as a mediating factor between AF and increased PTH concentrations. Primary aldosteronism is associated with an increased risk of AF (25), and mineralocorticoid receptor antagonism reduces AF incidence in heart failure populations (24). Intriguingly, aldosteronism is associated with urinary calcium wasting accompanied by secondary hyperparathyroidism (6, 26, 27). While experimental data suggested that aldosterone-induced secondary hyperparathyroidism may contribute to cardiomyocyte calcium overload, human interventional data demonstrated a dynamic relationship between the renin–angiotensin–aldosterone system and parathyroid hormone, supporting a clinically relevant interaction between RAAS activation and mineral metabolism (25, 28). Considering that upregulated activity of aldosterone is a key feature in the development of AF and HF, these studies raise the speculative hypothesis that PTH could represent one pathway within aldosterone-mediated cardiac remodeling, which warrants prospective evaluation (6).

The subgroup analyses presented in Table 5 should be interpreted with caution. The heterogeneity of results across subgroups – with a significant PTH–AF association observed in patients with arterial hypertension and in those with higher FGF23 concentrations, but not consistently across all strata – may reflect differential confounding by structural heart disease, medication burden, and overall cardiovascular risk rather than true biological effect modification. Notably, the significant associations in the hypertension and high-FGF23 strata were accompanied by wide confidence intervals, so that the apparent heterogeneity may partly reflect the limited precision of the reduced subgroup samples. Reassuringly, when the cohort was stratified by median age, the PTH–AF association remained significant in both the younger and older subgroups with overlapping confidence intervals; together with the inclusion of age as a covariate in all models, this argues against age as the primary driver of the association or of the observed subgroup heterogeneity. No formal tests for interaction were performed, and these analyses were pre-specified as exploratory; accordingly, the findings should not be overinterpreted. The attenuation of the association in certain subgroups may indicate that in patients with advanced cardiovascular remodeling and neurohumoral activation, established risk factors for AF dominate, thereby reducing the apparent independent contribution of PTH. Prospective data from the large, community-based ARIC study did not demonstrate an independent association between PTH and incident AF (29). This discrepancy likely reflects fundamental cohort differences: LURIC comprised high-risk patients referred for coronary angiography (14), whereas ARIC enrolled a lower-risk general population in whom PTH was less closely tied to cardiac and renal dysfunction. Moreover, the cross-sectional, prevalent-AF design of the present study differs from the incident-AF design of AF, so that the divergent findings may reflect effect modification by underlying cardiovascular risk rather than a true absence of association.

**Table 5:** Subgroup analysis.

Variable	Subgroup	AF (% of group)	OR (95% CI)	*p*-value
Arterial hypertension	No arterial hypertension	158 (39.8%)	1.56 (0.57–4.31)	0.388
	Arterial hypertension	239 (60.2%)	2.83 (1.31–6.11)	0.008
Age	Above median	270 (71.4%)	6.79 (3.62–12.74)	<0.001
	Below median	108 (28.6%)	3.36 (1.29–8.77)	0.013
FGF23	Below median	113 (32.1%)	2.55 (0.81–8.09)	0.111
	Above median	239 (67.9%)	2.22 (1.07–4.62)	0.033
Aldosterone	Below median	179 (48.2%)	2.13 (0.88–5.13)	0.093
	Above median	192 (51.8%)	2.32 (0.92–5.85)	0.075

Results of the multivariate binary logistic regression analysis with atrial fibrillation (AF) as the dependent variable in subgroups defined by history of arterial hypertension, below or above median age, below or above median FGF23, and serum aldosterone levels above or below median. PTH was log10-transformed prior to analysis, odds ratios for AF per log10-unit increase in PTH are presented. All models were adjusted according to Model 2.

**Figure 2:**
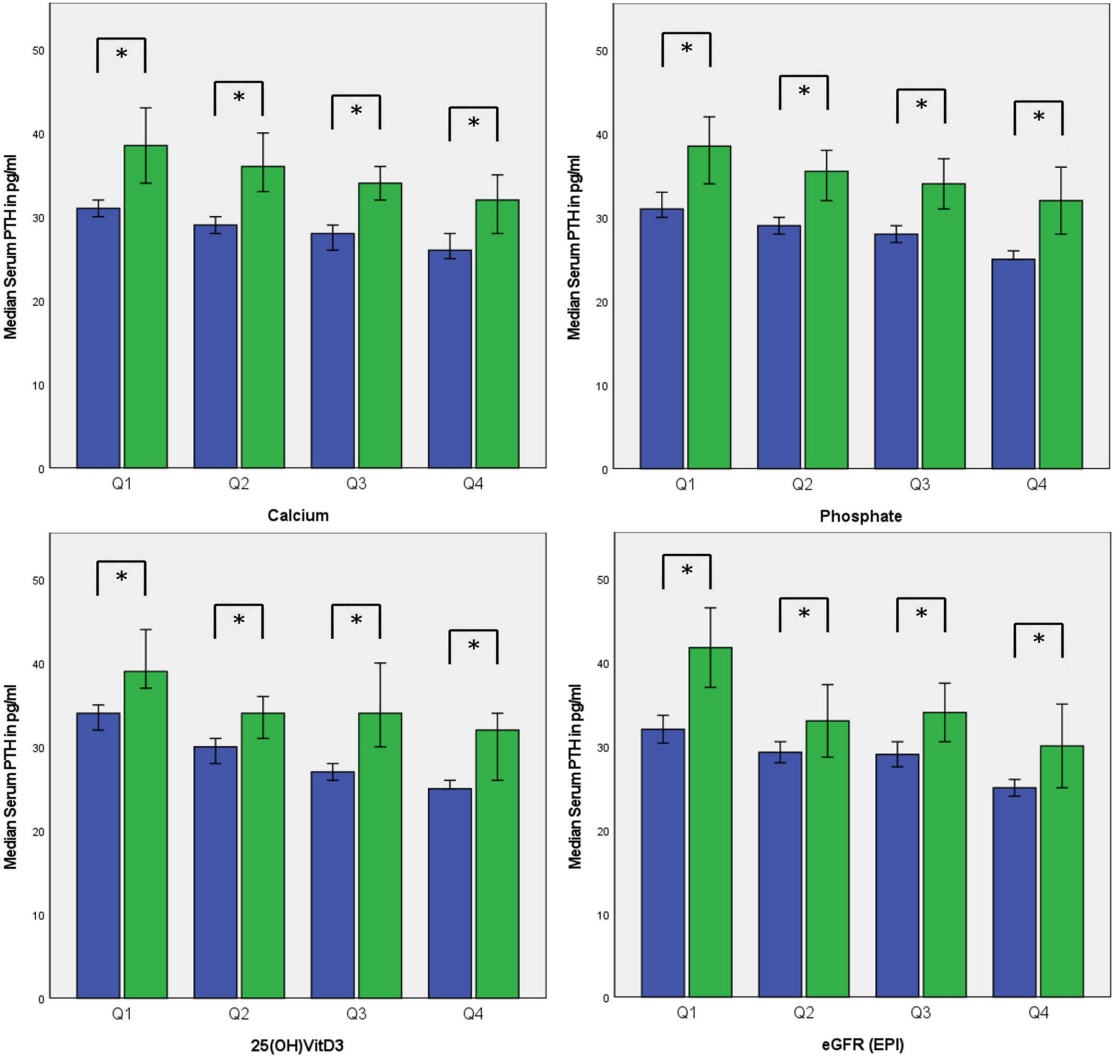
Serum parathyroid hormone (PTH) levels in patients with atrial fibrillation (green bars) vs. patients without atrial fibrillation (blue bars) according to quartiles of **(A)** serum calcium; **(B)** serum phosphate; **(C)** serum 25-hydroxy vitamin D_3_; **(D)** estimated glomerular filtration rate estimated according to the EPI formula. *p ≤ 0.05.

A further consideration is whether the PTH-AF association is confounded by medication or dysregulated calcium metabolism. Although digitalis and calcium antagonists acutely suppress PTH secretion, our multivariable models adjusting for both drugs yielded similar estimates (11), supporting an association independent of these pharmacological effects. PTH remained associated with AF across all quartiles of 25-hydroxy vitamin D_3_, eGFR, serum calcium and serum phosphate (Figure 2), arguing against the possibility that the observed association is solely driven by secondary hyperparathyroidism due to 25-hydroxy vitamin D_3_ deficiency or impaired kidney function. This is consistent with prospective data showing that neither 25-hydroxy vitamin D_3_deficiency nor reduced eGFR independently predict incident AF (30, 31). However, the biological plausibility of the FGF23 subgroup findings is supported by prior data identifying FGF23 as a predictor of atrial fibrillation. These exploratory findings are consistent with the hypothesis that broader alterations in mineral metabolism may contribute to AF risk, though the small subgroup sizes preclude firm conclusions. In the CRIC study, Mehta et al. demonstrated an association between elevated FGF23 levels and incident atrial fibrillation in patients with chronic kidney disease (32). These findings primarily apply to CKD populations; although the LURIC cohort shows impaired renal function, it does not constitute a formal CKD cohort.

Although left atrial diameter emerged as the dominant structural predictor of AF (Wald *χ*² = 67.8, *p* < 0.001), inclusion of this parameter together with NT-proBNP and NYHA class did not materially alter the association between PTH and AF. The modest attenuation (Effect estimates changed by <10% compared to Model 2) of the effect estimate suggests partial overlap with cardiac remodelling pathways, yet does not support the interpretation of PTH as a mere downstream marker of hemodynamic burden.

Beyond its association with AF, we investigated whether elevated PTH levels are also linked to stroke risk in this cohort. PTH was not associated with stroke in either univariable or multivariable Cox regression analysis, nor in the subgroup of AF patients. This null finding is consistent with results from the larger prospective ARIC and ARIC MRI studies, in which PTH was not independently associated with incident stroke after adjustment for 25-hydroxy vitamin D_3_, renal function and other confounders (29, 33). Nevertheless, the vascular biology of PTH warrants consideration, experimental evidence suggests that PTH may promote endothelial dysfunction through calcium overload and reactive oxygen species production, though the clinical relevance of these findings in humans at physiological PTH concentrations remains uncertain (15). Furthermore, elevated PTH has been associated with subclinical and clinical atherosclerotic disease (16). Whether PTH at the levels observed in the present cohort – within or only slightly above the normal range – is sufficient to confer measurable cerebrovascular risk remains to be determined in prospective studies with adequate event rates.

## 5 Limitations

Several limitations warrant consideration. First, due to the cross-sectional design, temporal relationships between PTH and AF cannot be determined, and causal inference is not possible. Although the association between PTH and AF remained robust after extensive adjustment, residual confounding cannot be excluded. Second, as the study population consisted of patients referred for coronary angiography in the late 1990s, representing a high-risk and highly selected cohort, findings may not be generalizable to contemporary, lower-risk or community-based AF populations. Third, although we performed multivariable adjustment for a wide range of clinical and biochemical variables, substantial differences in baseline characteristics between AF and non-AF patients raise the possibility of residual confounding. In particular, more refined measures of atrial size, atrial fibrosis, or hemodynamic load were not available, the invasive subset remained small although a rather large fraction of AF patients could be assessed. Furthermore, LVEF was visually estimated and may be subject to measurement variability. Fourth, adjustment for medication use may introduce bias, as several treatments included in the models are commonly prescribed as a consequence of AF rather than preceding it. Similarly, the structural and neurohumoral markers used in the sensitivity analyses (left atrial diameter, NT-proBNP, NYHA class, pulmonary pressures) may act as mediators rather than confounders, so that their adjustment risks overadjustment and the corresponding estimates should be interpreted as conservative. Finally, stroke analyses were limited by the modest number of events and restricted follow-up duration.

## 6 Conclusions

In conclusion, elevated serum PTH concentrations were associated with prevalent AF in a large cohort of patients undergoing coronary angiography, and this association persisted after adjustment for established AF risk factors and parameters of mineral metabolism. The persistence of the association after extensive adjustment suggests that the relationship is not entirely attributable to measured markers of cardiac dysfunction; however, residual confounding and overadjustment cannot be excluded. Whether PTH plays a contributory role in AF pathogenesis, acts as a mediator within the aldosterone–PTH axis, or primarily reflects hemodynamic load cannot be resolved from cross-sectional data and remains speculative. Prospective longitudinal studies with serial PTH measurements, comprehensive hemodynamic phenotyping, and incident AF as the primary endpoint are needed to clarify the temporal relationship and to determine whether modulation of mineral metabolism has clinical relevance in the context of AF.

## Data Availability

The data analyzed in this study is subject to the following licenses/restrictions: the underlying data for this analysis stemming from the LURIC study will be provided upon reasonable request. Requests to access these datasets should be directed to winfried.maerz@luric.online.
